# Prevalence and effect of attention-deficit/hyperactivity disorder on school performance among primary school pupils in the Hohoe Municipality, Ghana

**DOI:** 10.1186/s12991-017-0135-5

**Published:** 2017-02-14

**Authors:** Kingsley Afeti, Samuel Harrenson Nyarko

**Affiliations:** Department of Population and Behavioural Sciences, School of Public Health, University of Health and Allied Sciences, Hohoe, Ghana

**Keywords:** Prevalence, Attention-deficit/hyperactivity disorder, Primary school pupils, Hohoe, Ghana

## Abstract

**Background:**

Attention-deficit/hyperactivity disorder (ADHD) is one of the most common disorders in early childhood. However, not many studies have been conducted on the prevalence and effect of ADHD on school performance in Ghana. This study sought to ascertain the prevalence of ADHD and its effect on school performance among primary school pupils in the Hohoe municipality of Ghana.

**Methods:**

This is a cross-sectional descriptive study that included 400 primary school pupils in the Hohoe Municipality of Ghana. The study adopted the disruptive behaviour disorder rating scale which includes the three subtypes of ADHD among pupils in the form of a close-ended questionnaire for data collection.

**Results:**

The results revealed the overall prevalence of ADHD to be 12.8%. The males had a higher prevalence (14.4%) compared to the females (10.5%). For the subtypes, the prevalence was 8.0% for attention-deficit disorder, 8.5% for hyperactivity disorder and 3.8% for the combined subtype. In terms of school performance, the results showed that there was a significant difference in the school performance between ADHD-positive pupils and the negative status pupils among the various core subjects.

**Conclusions:**

Attention-deficit/hyperactivity disorder was quite prevalent among primary school pupils in the Hohoe Municipality, and has impacted negatively on their school performance. Screening of pupils for ADHD should be integrated into the school health services to enable early detection and management.

## Background

Education is an important tool for national development. In the developing countries including Africa, where the literacy level is low, significant efforts are directed toward the need to increase the level of literacy through increasing priority for education [1]. In Ghana, there have been some initiatives to improve upon school work, including the introduction of the School Feeding Programme, provision of school uniforms and shoes to students and the awarding of scholarships to needy but brilliant children. In spite of these efforts, little is done to identify and eliminate the impediments to learning, especially among children. One of such impediments is attention-deficit/hyperactivity disorder (ADHD), which has been identified to affect 3–12% of primary school children [2].

Attention-deficit/hyperactivity disorder is one of the most common disorders in early childhood [3]. It is characterized by inattention, hyperactivity, and impulsivity, cognitive, behavioural, and emotional deficits. It is also closely related to learning disabilities, lack of self-control, and social skill deficits [4]. There are three subtypes of ADHD: predominantly inattentive type (attention-deficit disorder, ADD), predominantly hyperactive type (hyperactivity disorder) and the combined type [5]. It has been estimated that more than 50% of children with ADHD are prone to experience other psychiatric disorders such as oppositional defiant disorder (ODD), depression and anxiety disorder [6]. The major etiological factor has been identified to be genetic [7].

While it was previously thought that children eventually outgrow ADHD, recent studies suggest that 30–60% of affected children continue to show significant symptoms of the disorder during adulthood [8]. ADHD can be counted as a major public health issue due to its prevalence and chronic nature, and its potential to interfere with different areas of developmental relevance [9]. Also, ADHD has effects on the school performance of children. Children with more symptoms of ADHD, including impulsiveness or restlessness, have significantly lower math and reading scores on standardized tests, increased probability of class repetition, enrollment in special education, and delinquency, which includes behaviours such as stealing, hitting people, or using drugs [10]. In adulthood, problems caused by ADHD may include lateness, absenteeism, excessive errors, and an inability to accomplish expected workloads. At home, relationship difficulties and breakups may be more common [11].

In spite of the fact that ADHD hinders school development in children and has adulthood negative effects, not much has been done on it. Little information also exists on the prevalence of ADHD and its effects on school children in Ghana. This study, therefore, sought to investigate the prevalence of ADHD and its effect on school performance by comparing the school performance of children diagnosed with ADHD and those without ADHD in the core subjects.

## Methods

The study was conducted in the Hohoe Municipality of the Volta Region, Ghana. It was conducted among pupils in 10 public and private schools between the ages of 6 and 12 in the classes of primary 1 to 6. This target population had no pupils with intellectual disability and their culturally compatible IQ tests would place them in the normal range of intelligence. The study adopted a descriptive cross-sectional study design.

Simple random sampling (lottery method) was used to select ten schools for the study. The process was carried out by writing the names of all the primary schools in the municipality on sheets of paper that were folded and then randomly picking 10 out of them. Quota sampling technique was then used to select 400 respondents from all the classes in the schools selected.

This study used the parent/teacher disruptive behaviour disorder rating scale (DBDRS) in the form of a close-ended questionnaire for data collection. This explores the prevalence of all three subtypes of ADHD among the pupils. The parent/teacher DBDRS is a standard rating scale used to screen children for the presence of ADHD and other disruptive behaviours diagnosed in early childhood. This rating scale was completed by the class teacher because it was assumed that the teacher might have been with the child for more than 3 months and, therefore, knew the child well enough to complete the rating scale on his or her behalf. The school performance of each pupil in the core subjects such as Mathematics, Science and English was also obtained from the school registers. The performance was, therefore, classified based on a grading of 70–100% for good performance, 40–69 for average and 39 or below for poor performance.

The completed questionnaires were cleaned and checked for completeness. Stata version 12 was used to process the data. The results were presented in tables in the form of frequencies, percentages and proportions, while Chi square test was used to examine the effect of ADHD on school performance of the pupils. Pupils diagnosed with the disorder were labelled ‘positive’ while those without the disorder were labelled ‘negative’.

## Results

### Background characteristics

A summary of the background characteristics of respondents has been presented in Table 1. The respondents were made up of 57.3% males and 42.7% females. The ages of the respondents ranged from 6 to 12 with an average age of 9.6. The highest representation of 40.5% was for respondents who were aged 11–12, while the least representation was for respondents aged 6–7 with 21.5%. The respondents were selected from classes 1–6 in primary schools. Class 1 was the most represented with 23.0%, followed by class 2 with 18.7%, while class 6 was the least represented with 11.5%.

**Table 1:** Background characteristics of respondents

Variables	Frequency	Percentages
*Sex*		
Male	229	57.3
Female	171	42.7
*Age*		
6–7	86	21.5
8–10	152	38.0
11–12	162	40.5
*Classes*		
1	92	23.0
2	75	18.7
3	64	16.0
4	61	15.3
5	62	15.5
6	46	11.5

### Prevalence of ADHD

The total prevalence of ADHD has been presented in Table 2. Out of the 400 respondents who participated in the study, 51 met the criteria for the diagnosis of ADHD, which represents a total prevalence of 12.8%. The prevalence is presented as the prevalence for males (14.41%), the prevalence for females (10.53%) and the total prevalence (12.8%).

**Table 2:** Total prevalence of attention-deficit/hyperactivity disorder

Variables	Negative	Positive	Total
*Sex*			
Male	196 (85.59%)	33 (14.41%)	229
Female	153 (89.47%)	18 (10.53%)	171
Total	349 (87.25%)	51 (12.75%)	400

Results were also presented for the various subtypes of ADHD by the background characteristics of the respondents. The results for the first subtype, ADD, have been presented in Table 3. As indicated in the table, the total prevalence of the ADD subtype was 8.0%, the male and female prevalence were 9.6% and 5.9%, respectively. However, there was no significant association between sex of respondent and ADD. In terms of age, the prevalence for respondents aged 8–10 was 10.5% while that for respondents aged 6–7 was 4.7%, though there was no significant association between age of respondent and ADD. With regard to the classes, the highest prevalence was found in class four (14.7%), followed by class two (12.0%), and the least was class one (2.2%). In effect, there was a significant association between class of respondent and the ADD subtype (PV = 0.034).

**Table 3:** Prevalence of the attention-deficit disorder subtype

Prevalence of attention-deficit disorder (inattention symptoms)				Chi square (*P* value)
	Negative ADHD status	Positive ADHD Status	Total	
	*N* = 368 (%)	*N* = 32 (%)	*N* = 400 (%)	
*Sex*				
Male	207 (90.4)	22 (9.6)	229 (57.3)	1.87 (0.170)
Female	161 (94.1)	10 (5.9)	171 (42.7)	
*Age*				
6–7	82 (95.3)	4 (4.7)	86 (21.5)	2.70 (0.258)
8–10	136 (89.5)	16 (10.5)	152 (38.0)	
11–12	150 (92.6)	12 (7.4)	162 (40.5)	
*Class*				
Class one	90 (97.8)	2 (2.2)	92 (23.0)	12.09 (0.034)
Class two	66 (88.0)	9 (12.0)	75 (18.8)	
Class three	59 (92.2)	5 (7.8)	64 (16.0)	
Class four	52 (85.3)	9 (14.7)	61 (15.2)	
Class five	60 (96.8)	2 (3.2)	62 (15.5)	
Class six	41 (89.1)	5 (10.9)	46 (11.5)	

A summary of the results for the second subtype, hyperactivity disorder (HD), is also presented in Table 4. The total prevalence of the HD subtype was 8.5%, the male and female HD prevalence were 9.6% and 7.0%, respectively, even though there was no significant association between sex of respondent and the HD subtype.

**Table 4:** Prevalence of the hyperactivity disorder subtype

Prevalence of hyperactivity disorder (hyperactivity/impulsivity symptoms)				Chi square (*P* value)
	Negative ADHD status	Positive ADHD status	Total	
	*N* = 366 (%)	*N* = 34 (%)	*N* = 400 (%)	
*Sex*				
Male	207 (90.4)	22 (9.6)	229 (57.3)	0.84 (0.358)
Female	159 (93.0)	12 (7.0)	171 (42.7)	
*Age*				
6–7	78 (90.7)	8 (9.3)	86 (21.5)	5.20 (0.074)
8–10	145 (95.4)	7 (4.6)	152 (38.0)	
11–12	143 (88.3)	19 (11.7)	162 (40.5)	
*Class*				
Class one	90 (97.8)	2 (2.2)	92 (23.0)	17.32 (0.004)
Class two	62 (82.7)	13 (17.3)	75 (18.8)	
Class three	62 (96.9)	2 (3.1)	64 (16.0)	
Class four	56 (91.8)	5 (8.2)	61 (15.2)	
Class five	57 (91.9)	5 (8.1)	62 (15.5)	
Class six	39 (84.8)	7 (15.2)	46 (11.5)	

For age, the prevalence of the HD subtype was highest (11.7%) among respondents aged 11–12, but was lowest among respondents aged 8–10 (4.6%). However, there was no significant association between age of respondent and the HD subtype. With the classes, the highest (17.3%) prevalence was among class two pupils, followed by class six pupils (15.2%), while the least was observed among class one pupils (2.2%). As such, the results showed a significant association between class of respondent and the HD subtype (PV = 0.004).

Table 5 shows a summary of the results for the combined subtype. The total prevalence of the combined type observed was 3.8%, while prevalence for males was 4.8% and that for females was 2.3%. However, no significant association was observed between sex of respondent and the combined subtype. The highest prevalence of 4.0% was observed among pupils aged 8–10, while the lowest prevalence (3.5%) was observed among pupils aged 6–7, and there was no significant association between age of respondent and the combined subtype. Disparities were also observed among the different classes with the highest (6.7%) prevalence being among class two pupils and the least being among classes two and five pupils (1.6%), though no significant association was observed between class of respondent and the combined subtype.

**Table 5:** Prevalence of the combined subtype

Combined subtype				Chi square (*P* value)
	Negative ADHD status	Positive ADHD status	Total	
	*N* = 385 (%)	*N* = 15 (%)	*N* = 400 (%)	
*Sex*				
Male	218 (95.2)	11 (4.8)	229 (57.3)	1.64 (0.199)
Female	167 (97.7)	4 (2.3)	171 (42.7)	
*Age*				
6–7	83 (96.5)	3 (3.5)	86 (21.5)	0.03 (0.983)
8–10	146 (96.0)	6 (4.0)	152 (38.0)	
11–12	156 (96.3)	6 (3.7)	162 (40.5)	
*Class*				
Class one	90 (97.8)	2 (2.2)	92 (23.0)	5.24 (0.387)
Class two	70 (93.3)	5 (6.7)	75 (18.8)	
Class three	63 (98.4)	1 (1.6)	64 (16.0)	
Class four	58 (95.1)	3 (4.9)	61 (15.2)	
Class five	61 (98.4)	1 (1.6)	62 (15.5)	
Class six	43 (93.5)	3 (6.5)	46 (11.5)	

### Effect of ADHD on school performance of pupils with ADHD

The study also sought to examine the possible effect of ADHD on the school performance of the respondents. Table 6, therefore, shows a summary of results on the performance of ADHD-positive respondents as compared to respondents who were ADHD negative. As indicated in the table, close to half (49.2%) of the respondents had an average performance in Mathematics and 42% had good performance while only 8.8% had poor performance. However, 26.7% of the ADHD positive pupils performed poorly compared to 8.1% of the ADHD-negative pupils. Also, 13.3% of the positive pupils had a good performance compared to 43.1% of the ADHD negative respondents. Consequently, the results showed a significant difference in performance in Mathematics between respondents diagnosed with ADHD and those without ADHD (PV = 0.010).

**Table 6:** School performance of pupils with the ADHD combined subtype

School performance of students with ADHD combined type				Chi square (*P* value)
	Negative ADHD status	Positive ADHD status	Total	
	*N* = 385 (%)	*N* = 15 (%)	*N* = 400 (%)	
*Mathematics*				
Poor performance	31 (8.1)	4 (26.7)	35 (8.8)	9.13 (0.010)
Average performance	188 (48.8)	9 (60.0)	197 (49.2)	
Good performance	166 (43.1)	2 (13.3)	168 (42.0)	
*Science*				
Poor performance	33 (8.6)	3 (20.0)	36 (9.0)	8.52 (0.014)
Average performance	186 (48.3)	11 (73.3)	197 (49.3)	
Good performance	166 (43.1)	1 (6.7)	167 (42.7)	
*English*				
Poor performance	33 (8.6)	2 (13.3)	35 (8.8)	12.22 (0.002)
Average performance	177 (46.0)	13 (86.7)	190 (47.5)	
Good performance	175 (45.4)	0 (0.0)	175 (43.7)	

For science, 49.3% of the respondents performed averagely, 42% had good performance, and only 9.0% performed poorly. Twenty percent of the ADHD-positive respondents had poor performance compared to 8.6% of the ADHD-negative respondents. Likewise, 6.7% of the ADHD-positive respondents attained good performance compared to 43.1% of the ADHD-negative respondents. Therefore, the study found a significant difference in performance in the Science subject between ADHD-positive and the ADHD-negative respondents (PV = 0.014).

With regard to English language, 47.5% of the respondents were average performers while 43.7% were good performers with only 8.8% being poor performers. About 13% of the ADHD-positive respondents performed poorly compared to 8.6% for the ADHD-negative respondents who performed poorly. In the same way, none of the ADHD-positive respondents were good performers in English language compared to 45.4% of the ADHD-negative respondents who were good performers in the subject. The study, therefore, showed a significant difference in performance in the English language subject between the ADHD-positive and the negative respondents (PV = 0.002).

## Discussion

This study found the total prevalence of ADHD among pupils in the Hohoe municipality to be 12.8%. In the context of the study area, this prevalence may be on the high side. On the other hand, a similar study also came out with a similar prevalence of 11.6% in Jeddah City, Saudi Arabia [12]. However, the prevalence of ADHD in this study is rather quite lower than the prevalence in other studies such as the 23.1% found in Benin Metropolis, Nigeria by Abikwi [13].

Furthermore, the findings of the study showed prevalence of the subtypes as ADD 8.0%, HD 8.5%, and the combined subtype of 3.8%. In a similar study conducted in Lebanon, Bathiche [14] found the prevalence of the subtypes of ADHD to be 11.4% for the ADD (inattentive), 8.7% for the HD (the hyperactive/impulsive), and 3.5% for the combined type. However, these results are quiet higher compared to findings from other studies such as Ofovwe et al. [15], where the prevalence of the various subtypes were 3.0% for ADD, 2.7% for HD, and 2.5% for the combined subtype. This implies that the prevalence of ADHD varies among studies due to a number of factors, some of which may be the study setting as well as the target population of the study.

The study showed a significantly higher prevalence among males compared to females in all the three subtypes of ADHD, making it a common disorder among the males than the females. Also, the results showed that sex and age of respondent had no association with all the three subtypes of the disorder. This could mean that the symptoms may not differ between the sexes and may not diminish as the child grows older. However, class of respondent had a significant association with two of the subtypes except the combined subtype. This may imply that regardless of the child’s sex or age, there were some significant differences among the various classes in terms of the ADD and the HD. It may also mean that the prevalence or the symptoms of ADD and HD may persist with the increase in the class or age of the respondents.

The results further revealed that school performance in subjects such as Mathematics, Science and English was poorer among ADHD-positive respondents than the ADHD-negative respondents. Thus, not only was the school performance poor among the ADHD-positive respondents compared to the ADHD-negative respondents, but also, there were significant differences in the school performance of the two categories of respondents in all the three subjects. This means that ADHD has serious implications for the school performance of the respondents. A number of similar studies around the globe have, therefore, come out with similar findings. For instance, Daley and Birchwood [16] have come out that ADHD is associated with school underachievement across the developmental spectrum, from preschoolers to adults. In another study, Bolic et al. [17] indicate that students with ADHD had low satisfaction with computer use in school, which implied that pupils with ADHD are more likely to have poorer school performance in educational activities that require the use of computer than their counterparts. This may be explained as a result of lack of focus on school work due to the various obvious symptoms of the disorder among the respondents. However, a potential limitation of this study may be that since the class teacher is the only observer and respondent of the rating scale, it is possible for the teacher to be biased, which could influence the findings particularly at the lower classes. Nevertheless, this may not have any direct effect on the findings, since it has to do with school performance of pupils and not children in general.

## Conclusions

The study showed that ADHD is quite a prevalent disorder among primary school pupils in the Hohoe Municipality, Ghana. The disorder has a dire implication for the school performance of the school pupils in the municipality. Hence, pupils with positive ADHD status are more likely to perform poorly in school compared to their counterparts with negative ADHD status. It is, therefore, imperative to integrate the screening of pupils for ADHD into the school health services in order to enable early detection and management of the disorder. Also, there is the need to intensively educate teachers on ADHD and how they can manage these pupils in order to help to improve their school performance. This can be done by the District Health Directorate through organizing workshops on ADHD for teachers.

## Abbreviations

ADHD: attention-deficit/hyperactivity disorder
ADD: attention-deficit disorder
HD: hyperactivity disorder
DBDRS: disruptive behaviour disorder rating scale
IQ: intelligent quotient
